# Luteinizing Hormone Suppression by Progestin-Primed Ovarian Stimulation Is Associated With Higher Implantation Rate for Patients With Polycystic Ovary Syndrome Who Underwent *in vitro* Fertilization/Intracytoplasmic Sperm Injection Cycles: Comparing With Short Protocol

**DOI:** 10.3389/fphys.2021.744968

**Published:** 2022-02-11

**Authors:** Chen Chen, Sha Yu, Weina Yu, Zhiguang Yan, Wei Jin, Jiqiang Si, Menghui Li, Renfei Cai, Dongying Li, Li Wang, Qiuju Chen, Yanping Kuang, Qifeng Lyu, Hui Long

**Affiliations:** Department of Assisted Reproduction, Shanghai Ninth People’s Hospital, Shanghai Jiao Tong University School of Medicine, Shanghai, China

**Keywords:** luteinizing hormone, polycystic ovary syndrome, progestin-primed ovarian stimulation, implantation rate, oocyte

## Abstract

**Background:**

Many studies have demonstrated the positive clinical value of progestin-primed ovarian stimulation (PPOS) in patients with polycystic ovary syndrome (PCOS) who underwent assisted reproductive technology. However, the underlying factors contributing to this phenomenon remain unclear. We conducted a retrospective observational study to compare the clinical outcomes of women with PCOS who underwent PPOS or the short protocol to identify possible factors that influence the outcome.

**Methods:**

This study included 304 patients who underwent PPOS and 152 patients who underwent short protocol from April 2014 to July 2019 after propensity-score matching. Human menopausal gonadotropin (hMG) dose, hormone profile, embryo development, and clinical outcomes of frozen-thawed embryo transfer (FET) cycles were compared. The primary outcome measure was the implantation rate. Logistic regression was performed to identify contributing factors, and receiver operating characteristic curve analysis was used to calculate the cutoff of luteinizing hormone (LH) difference ratio in clinical outcomes.

**Results:**

Compared with the short protocol, PPOS resulted in a higher implantation rate (43.4% vs. 31.9%, *P* < 0.05), clinical pregnancy rate (61.8% vs. 47.4%, *P* < 0.05), and live birth rate (48.4% vs. 36.8%, *P* < 0.05). Similar fertilization, cleavage, and valid embryo rate per oocyte retrieved between groups were observed. The LH difference ratio was positively associated with implantation rate [*P* = 0.027, odds ratio (OR) = 1.861, 95% CI: 1.074–3.226]. The relationship between the LH difference ratio with clinical outcomes was confirmed by receiver operating characteristic curve analysis and comparisons among patients grouped by the LH difference ratio.

**Conclusion:**

The implantation rate was associated with the LH difference ratio during ovary stimulation in patients with PCOS. Our results provide the explanation why PPOS shows the positive clinical outcomes for patients with PCOS.

## Introduction

Polycystic ovary syndrome (PCOS) is the most common endocrinopathy in women of reproductive age, and more than 80% of patients with anovulatory infertility suffer from PCOS (Thessaloniki EA-SPCWG, 2008; Balen et al., 2016). *In vitro* fertilization (IVF) is the final reproductive therapy strategy for patients with PCOS who did not achieve benefit from medical treatment and ovarian surgery (Azziz et al., 2016). However, oocytes retrieved from patients with PCOS are typically of poor quality, leading to lower fertilization and cleavage rates, and producing lower quality embryos with a low implantation rate during IVF treatment (Qiao and Feng, 2011).

Progestin-primed ovarian stimulation (PPOS) is a novel ovarian stimulation method for IVF that uses oral progestin as an alternative to gonadotropin-releasing hormone analogs (Kuang et al., 2015; Massin, 2017; Ata et al., 2020). Studies by our group and others showed that PPOS effectively prevents the premature luteinizing hormone (LH) surge and leads to positive clinical outcomes in patients with PCOS (Wang et al., 2016; Zhu et al., 2016; Sun et al., 2018; Xiao et al., 2019; Ata et al., 2020; Gurbuz and Gode, 2020) and in patients with non-PCOS (Ata et al., 2020; La Marca et al., 2020; Martinez et al., 2020). Previous randomized and retrospective studies reported optimal clinical outcomes, such as higher rates of fertilization, pregnancy, and implantation, in patients with PCOS treated with PPOS (Wang et al., 2016; Zhu et al., 2016). However, the mechanism by which PPOS improves IVF outcomes in patients with PCOS is still poorly understood.

Although treatments that induce ovulation can overcome the inability of patients with PCOS to ovulate, the high rate of pregnancy loss is another issue for these patients (Qiao and Feng, 2011; Azziz et al., 2016). The reasons for the increased frequency of loss of pregnancy are not completely clear, but one possible explanation is the compromised oocyte competence resulting from an abnormal endocrine environment through maturation (Palomba et al., 2017). The classic endocrine abnormality in PCOS is the hypersecretion of LH, with a hyperactive gonadotropin-releasing hormone neural circuit and defects in progestin and estradiol feedbacks (Moore and Campbell, 2017; Coutinho and Kauffman, 2019). Accumulating evidence has shown that tonic hypersecretion of LH negatively impacts the outcome of assisted reproductive technology, with impaired pregnancy rates and higher miscarriage rates (Stanger and Yovich, 1985; Regan et al., 1990; Balen et al., 1993; Qiao and Feng, 2011). However, progestin was shown to suppress the LH surge and tonic LH secretion in animal models (Dierschke et al., 1973; He et al., 2017; Ata et al., 2020). This progestin-induced LH suppression was also observed in patients who underwent PPOS (Soules et al., 1984; Kuang et al., 2015; Massin, 2017). Therefore, we hypothesized that there may be a relationship between the decrease of LH level induced by progestin and the improved clinical outcomes in patients with PCOS treated with PPOS.

In this study, we compared the embryo development, implantation potential and subsequent clinical outcomes in women with PCOS who underwent different stimulation protocols and examined the underlying factors that may have an influence on the outcomes.

## Materials and Methods

### Study Design and Patients

This retrospective study was conducted at the Department of Assisted Reproduction at the Shanghai Ninth People’s Hospital, Shanghai Jiao Tong University School of Medicine, a tertiary research and education hospital. Women with PCOS who underwent IVF/intracytoplasmic sperm injection (ICSI) cycles with stimulation of PPOS or short protocol from April 2014 to July 2019 were reviewed. Eligible participants were 20–40 years old with a history of infertility of over 1 year. PCOS was diagnosed according to the Rotterdam consensus by meeting two out of the three following criteria: (1) oligo- and/or anovulation; (2) biochemical and/or clinical evidence of hyperandrogenism; and (3) polycystic ovarian morphology on ultrasound (Rotterdam EA-SPCWG, 2004). Patients with other etiologies of ovulatory dysfunction and hyperandrogenism were excluded, such as hyperprolactinemia, congenital adrenal hyperplasia, androgen-secreting tumors, and thyroid disease.

Patients were excluded if they met one of the following criteria: (1) fresh transfer cycles; (2) age >40 years; (3) basal follicle-stimulating hormone (FSH) level ≥10 IU/L; (4) endometriosis grade 3 or higher; (5) history of ovarian surgery; (6) uterine anomalies; (7) history of recurrent spontaneous abortion; (8) abnormal chromosomal karyotype; (9) fetal reduction in the first frozen-thawed embryo transfer (FET) cycles; or (10) lost to follow-up. In cases of patients who underwent more than one IVF/ICSI cycle with the use of the same stimulation program, only the first IVF/ICSI cycle was included in the cohort group for propensity score matching (PSM).

This study was approved by the Institutional Review Board of the Shanghai Ninth People’s Hospital affiliated with Shanghai Jiao Tong University School of Medicine.

### Ovarian Stimulation and Laboratory Procedures

All patients were examined by transvaginal ultrasound screen and blood test to confirm the ovarian status and determine the baseline hormone profile on day 3 of the menstrual cycle (MC3). The PPOS protocol was administered as described previously (Wang et al., 2016; Dong et al., 2017). In brief, human menopausal gonadotropin (hMG) at a dose of 150–225 IU/day (Fengyuan Pharmaceutical Co., Maanshan, China) and oral medroxyprogesterone acetate (MPA) 10 mg/day (Xianju Pharmaceutical Co., Taizhou, China) were administered daily from MC3 until the trigger day (Dong et al., 2017). In the short protocol group, patients received 0.1 mg of triptorelin (Ferring International Center SA, Germany) starting on MC2 and 150–225 IU of hMG daily starting on MC3 (Wang et al., 2016; Zhu et al., 2016).

In both groups, serum hormone concentrations and the number and size of developing follicles were measured every 2–4 days by ultrasound and blood examination from MC8–9. The hMG dose was adjusted based on the individual ovarian response and the dynamics of FSH, LH, and other hormones. When three dominant follicles reached 18 mm in diameter, the final stage of oocyte maturation was induced.

For the PPOS group, most patients with PCOS (*n* = 243) were triggered with both 0.1 mg triptorelin and 1,000 IU human chorionic gonadotropin (hCG, Lizhu Pharmaceutical Trading Co., Shanghai, China). A few patients with PCOS received 0.1 mg triptorelin (*n* = 42) or 2,000–3,000 IU hCG (*n* = 19). In the short protocol group, all patients were triggered with 2,000–3,000 IU hCG.

Oocyte retrieval was conducted 34–37 h after the trigger, guided by transvaginal ultrasound. The MPA dose was maintained at the same level and continued up to the trigger day.

The aspirated oocytes were fertilized via IVF or ICSI according to semen parameters (Henkel and Schill, 2003). Oocyte insemination was performed following standard procedures for IVF/ICSI (Salha et al., 1998; Rienzi et al., 2011). All embryos were cultured in separate microdroplets of a continuous single culture medium (Irvine Scientific, Santa Ana, CA, United States). Embryos were scored according to Cummins’s standard on day 3; high-quality embryos, which were defined as grade I or grade II, were selected for vitrification (Cummins et al., 1986). Non-high-quality embryos were subjected to extended culture until the blastocyst stage. Blastocysts with good morphology (grade ≥3 BC) were selected for cryopreservation (Gardner et al., 2000).

### Frozen-Thawed Transfer and Follow-Up of Clinical Outcomes

The detailed protocol for the endometrial preparation has been previously described (Wang et al., 2016; Huang et al., 2019). The distributions of patients receiving different endometrium preparation methods were similar between the study group and control group after PSM. In our center, viable embryos defined as blastocysts with good morphology or high-quality embryos after cleavage were suitable for embryo transfer. FET was performed by skilled physicians with the guidance of abdominal ultrasound. When pregnancy was achieved, luteal-phase support was continued until 10 weeks of gestation.

The follow-up system at our center was previously described (Zhu et al., 2016; Huang et al., 2019). The primary end point was the implantation rate, and the secondary outcome was the live birth rate. Other measurements included total hMG dose; FSH, LH, estradiol (E2), and progesterone (P) on trigger day; FSH difference ratio; LH difference ratio; normal fertilization rate; cleavage rate; valid embryo rate per oocyte retrieved; and biochemical pregnancy, ectopic pregnancy, clinical pregnancy, and miscarriage events after FET.

The FSH difference ratio was calculated as the difference between FSH on the trigger day and FSH on MC3 divided by FSH on MC3. The LH difference ratio was calculated as the difference between LH on MC3 and LH on the trigger day divided by LH on MC3. Normal fertilization rate was defined as the number of normally fertilized oocytes divided by the number of total retrieved metaphase II stage oocytes. Cleavage rate was defined as the number of zygotes cleaved divided by the number of normally fertilized oocytes. The viable embryo rate per oocyte retrieved was defined as the number of viable embryos divided by the number of oocytes retrieved. The implantation rate was defined as the number of gestational sacs observed in the uterus (excluding gestational sacs in patients with ectopic pregnancy) divided by the number of embryos transferred. Clinical pregnancy was defined as at least one gestational sac with or without fetal heart activity at 7 weeks after FET. Miscarriage was defined as the loss of clinical pregnancy before the gestational week 24. Live birth was identified as the delivery of at least one live baby after at least 24 weeks gestation.

### Statistical Analysis

Statistical analyses and PSM were performed using Statistical Package for the Social Sciences version 25.0 software (SPSS Inc., Chicago, IL, United States).

The normality of quantitative variables was tested by the Kolmogorov–Smirnov tests, Shapiro–Wilk tests, histograms, and Q-Q plots. Data are presented as the mean ± SD or medians (first quartile, third quartile) as appropriate. Comparison of between-group differences was performed with Student’s *t*-test or Mann–Whitney *U*-test. For qualitative variables, the Chi-square test or Fisher exact test was used to analyze the differences, and data are presented as % (n/N).

A PSM model was established using logistic regression. To balance significant differences between the two groups, 14 covariates were selected into the PSM model to estimate the propensity score, such as age (continuous), body mass index (BMI) (continuous), duration of infertility (continuous), gravidity (continuous), parity (continuous), infertility type (primary or secondary) and diagnosis (PCOS + tubal factor, PCOS + male factor, PCOS + mixed factors, or PCOS only/other factors), basal endocrine profiles (all continuous), antral follicle count (continuous), insemination method (IVF, ICSI, IVF + ICSI), endometrial thickness on FET day (continuous), endometrial preparation (mild stimulation, hormone replacement therapy), and the number and stage of embryos transferred (1 or 2, cleavage or blast, respectively). Patients with PCOS that received short protocol were matched with patients in the PPOS group using the nearest-neighbor random matching algorithm at a ratio of 1:2. All *P*-values were based on two-sided tests, and *P* < 0.05 was considered to indicate statistical significance.

## Results

### Patient Characteristics

**FIGURE 1:**
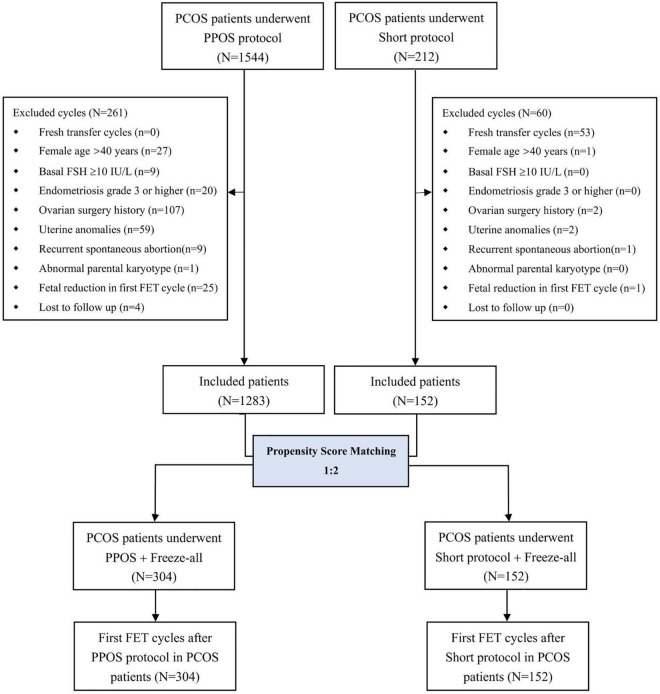
Flow chart of the current study. PCOS, polycystic ovary syndrome; PPOS, progestin-primed ovarian stimulation; FSH, follicle-stimulating hormone; FET, frozen-thawed embryo transfer.

A flow chart illustrating the study design with details on patient selection is shown in Figure 1. Briefly, a total of 1,756 female patients with PCOS who underwent IVF/ICSI were screened from our database, such as 1,544 treated with PPOS and 212 treated with the short protocol (Supplementary Table 1). After PSM, 152 patients who underwent the short protocol were matched with 304 patients treated with the PPOS protocol, and these 456 patients represented the final study group. All 456 participants successfully completed at least one FET cycle after oocyte retrieval and freeze-all cycles. No significant differences were found between the PPOS and short protocol groups in post-matching analysis with regard to characteristics of patients and the first FET cycle, such as age, BMI, duration of infertility, gravidity, parity, infertility type and diagnosis, basal endocrine profiles, antral follicle count, insemination method, endometrial thickness on FET day, endometrial preparation and the number and stage of embryos transferred (Supplementary Table 2). The standardized differences before and after matching are shown in Supplementary Figure 1. To decrease the potential bias, the first FET cycles were included after matching the characteristics described above.

### Patients Who Underwent Progestin-Primed Ovarian Stimulation Obtained a Higher Implantation Rate and Live Birth Rate Than Patients Treated With the Short Protocol

We compared the clinical outcomes of the total FET cycles from the 1,756 patients with PCOS (Supplementary Table 3) and found a higher implantation rate of embryos originating from patients receiving the PPOS protocol (42.2% vs. 31.4%, *P* < 0.05) and higher live birth rate per transfer (47.3% vs. 35.7%, *P* < 0.05, Supplementary Table 3) compared with patients treated with the short protocol.

In the comparison of the outcomes of the 456 matched patients, the implantation rate (43.4% vs. 31.9%), biochemical pregnancy rate (66.1% vs. 55.9%), and clinical pregnancy rate (61.8% vs. 47.4%) were all higher in the PPOS group than in the short protocol group (all *P* < 0.05, Table 1). These results indicate that the embryos from the PPOS group exhibited better implantation potential than those from the short protocol group. Notably, the live birth rate was also much better in the PPOS group (48.4% vs. 36.8%, *P* < 0.05). However, we observed a similar ectopic pregnancy rate (3.2% vs. 2.8%, *P* > 0.05) and miscarriage rate (18.6% vs. 19.4%, *P* > 0.05) between the two groups. In addition, the oocyte performance in the early developmental stage, such as the normal fertilization rate, cleavage rate, and valid embryo rate per oocyte retrieved, was similar between the two groups (*P* > 0.05, Supplementary Figure 2).

**TABLE 1:** Clinical outcomes of the first frozen-thawed embryo transfer cycle in patients with PCOS treated by PPOS or the short protocol after matching.

	PPOS (*n* = 304)	Short protocol (*n* = 152)	*P* value
**Implantation rate**	43.4 (243/560)	31.9 (90/282)	0.001
**Biochemical pregnancy rate**	66.1 (201/304)	55.9 (85/152)	0.034
**Clinical pregnancy rate**	61.8 (188/304)	47.4 (72/152)	0.003
**Ectopic pregnancy rate**	3.2 (6/188)	2.8 (2/72)	1.000
**Miscarriage rate**	18.6 (35/188)	19.4 (14/72)	0.879
**Live birth rate**	48.4 (147/304)	36.8 (56/152)	0.020
Singleton	34.5 (105/304)	27.6 (42/152)	
Multiple	13.8 (42/304)	9.2 (14/152)	

*Data are shown as % (n). PPOS, progestin-primed ovarian stimulation. The denominator for implantation rate is the number of embryos transferred. The denominator for ectopic pregnancy and miscarriage rate is the subjects of clinical pregnancies.*

### Comparison of Human Menopausal Gonadotropin Dose and Hormone Profiles in Ovarian Stimulation Cycles of Transferred Embryos

**FIGURE 2:**
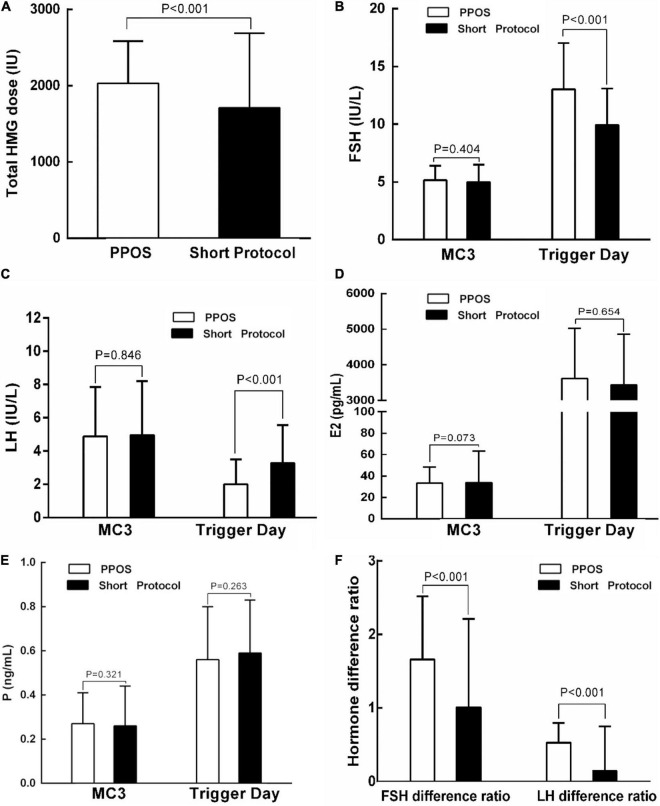
Comparison of the hMG dose and hormone profiles in ovarian stimulation cycles corresponding to the transferred embryos in patients with PCOS treated with indicated protocols. **(A)** Comparison of the hMG dose between the PPOS and short protocol groups. **(B–F)** Comparison of the FSH, LH, E2, P, or Hormone difference ratio between the PPOS and short protocol groups on MC3 or on trigger day. PPOS, progestin-primed ovarian stimulation; hMG, human menopausal gonadotropin; MC3, day 3 of the menstrual cycle; FSH, follicle-stimulating hormone; LH, luteinizing hormone; E2, estradiol; P, progesterone.

To identify the possible factors that may contribute to the difference in the embryo implantation potential between the PPOS and short protocol groups, we examined the hMG dose and hormone profile in stimulation cycles corresponding to the 842 embryos used in FET cycles. Compared with the short protocol group, the PPOS group used a higher dose of hMG (2031.88 ± 552.31 IU vs. 1709.38 ± 979.85 IU, *P* < 0.001, Figure 2A). Furthermore, the serum concentration of FSH increased markedly on the trigger day in the PPOS group (13.02 ± 4.01 IU/L vs. 9.93 ± 3.17 IU/L, *P* < 0.001, Figure 2B), and this may be from the higher dose of hMG used in the PPOS group. A higher FSH difference ratio was also observed in the PPOS group (1.66 ± 0.86 vs. 1.01 ± 1.20, *P* < 0.001, Figure 2F). However, the LH level on the trigger day was decreased significantly in the PPOS group (2.00 ± 1.51 vs. 3.28 ± 2.28, *P* < 0.001, Figure 2C), and a higher LH difference ratio was also observed (0.53 ± 0.27 vs. 0.15 ± 0.60, *P* < 0.001, Figure 2F). No significant difference was observed in E2 and P levels on the trigger day between the two groups (*P* > 0.05, Figures 2D,E).

To determine the relationship between the hMG dose and hormone levels with the implantation rate in the first FET cycle, we performed logistic regression analysis (Table 2). The independent variables included all factors that showed a significant difference between the two groups, such as total hMG dose, FSH on trigger day, LH on trigger day, FSH difference ratio, LH difference ratio, and ovulation trigger method (Figure 2). Logistic regression revealed a significant positive effect of the LH difference ratio on implantation success [*P* = 0.027, odds ratio (OR) = 1.861, 95% CI: 1.074–3.226]. The other independent variables did not reach statistical differences.

**TABLE 2:** Factors influencing implantation success in the first frozen-thawed embryo transfer cycle by logistic regression analysis.

	*P* value	OR	95% CI
**Total hMG dose**	0.968	1.000	1.000–1.000
**FSH on trigger day**	0.886	1.004	0.955–1.055
**LH on trigger day**	0.936	1.005	0.890–1.134
**FSH difference ratio**	0.905	0.984	0.761–1.273
**LH difference ratio**	0.027	1.861	1.074–3.226
**Ovulation trigger method**			
GnRH-a	**Reference**	**–**	**–**
HCG	0.939	0.972	0.473–1.999
Dual trigger	0.534	1.240	0.629–2.445

*hMG, human menopausal gonadotropin; FSH, follicle-stimulating hormone; LH, luteinizing hormone; FSH difference ratio = (FSH level on trigger day – FSH on MC3)/FSH level on MC3; LH difference ratio = (LH level on MC3 – LH on trigger day)/LH level on MC3; GnRH-a, gonadotrophin releasing hormone agonist; HCG, human chorionic gonadotrophin; CI, confidence interval.*

**FIGURE 3:**
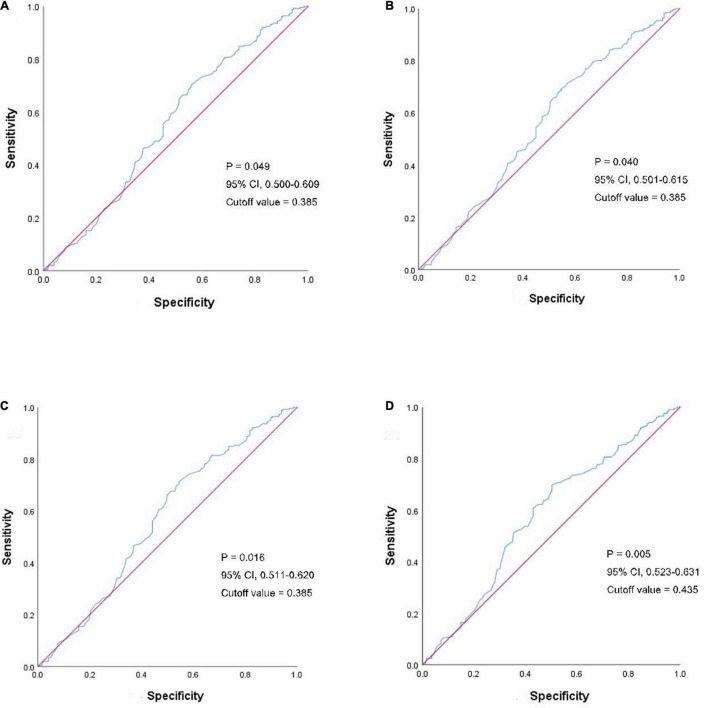
ROC analyses of the LH difference ratio in clinical outcomes. The blue line shows the LH difference ratio and the red line is the control. **(A)** ROC analyses of LH difference ratio in embryo implantation. **(B)** ROC analyses of LH difference ratio in a biochemical pregnancy. **(C)** ROC analyses of LH difference ratio in clinical pregnancy. **(D)** ROC analyses of LH difference ratio in the live birth. ROC, receiver operating curve; LH, luteinizing hormone.

To further study the LH difference ratio, we performed receiver operating curve (ROC) analysis to observe the influence in embryo implantation (Figure 3A, *P* = 0.049, 95% CI: 0.500–0.609, cutoff value = 0.385), biochemical pregnancy (Figure 3B, *P* = 0.040, 95% CI: 0.501–0.615, cutoff value = 0.385), clinical pregnancy (Figure 3C, *P* = 0.016, 95% CI: 0.511–0.620, cutoff value = 0.385), and live birth (Figure 3D, *P* = 0.005, 95% CI: 0.525–0.631, cutoff value = 0.435).

### Comparison of Clinical Outcomes in Patients With Polycystic Ovary Syndrome Stratified According to the Luteinizing Hormone Difference Ratio

Based on the cut-off value identified in ROC analysis, we divided the 456 patients with PCOS into two groups according to the LH difference ratio: the low ratio group (LH difference ratio <0.385) and the high ratio group (LH difference ratio ≥0.385). No significant difference in the endometrial thickness on FET day, endometrial preparation or the number or stage of embryos transferred was observed between the two groups (Supplementary Table 4).

We also evaluated the embryo implantation potential and subsequent clinical outcomes in the two groups and found that the implantation rate (43.0% vs. 33.2%), biochemical pregnancy rate (68.1% vs. 52.5%), clinical pregnancy rate (63.1% vs. 45.6%), and live birth rate (50.3% vs. 33.5%) were significantly higher in the high ratio group compared with the low ratio group (all *P* < 0.05, Table 3), indicating that patients with a high LH difference ratio showed improved outcome. Consistent with the previous analysis, the high ratio group had a higher percentage of patients treated with the PPOS protocol (PPOS and short protocol: 77.5% and 22.5%), and the low ratio group had a lower percentage (PPOS and short protocol: 46.2% vs. 53.8%), suggesting that the difference in the clinical outcome may be from the higher LH difference ratio induced by PPOS.

**TABLE 3:** Comparison of clinical outcomes in patients with PCOS stratified according to the LH difference ratio.

	Low ratio group (*n* = 158)	High ratio group (*n* = 298)	*P* value
**LH difference ratio**	<0.385	≥0.385	
**Percentage of ovarian stimulation**			<0.001
PPOS group	46.2 (73/158)	77.5 (231/298)	
Short protocol group	53.8 (85/158)	22.5 (67/298)	
**Implantation rate**	33.2 (99/298)	43.0 (234/544)	0.005
**Biochemical pregnancy rate**	52.5 (83/158)	68.1 (203/298)	0.001
**Clinical pregnancy rate**	45.6 (72/158)	63.1 (188/298)	<0.001
**Ectopic pregnancy rate**	1.4 (1/72)	3.7 (7/188)	0.566
**Miscarriage rate**	25.0 (18/72)	16.5 (31/188)	0.116
**Live birth rate**	33.5 (53/158)	50.3 (150/298)	0.001
Singleton	22.2 (35/158)	37.6 (112/298)	
Multiple	11.4 (18/158)	12.8 (38/298)	

*Data are shown as % (n). PPOS, progestin-primed ovarian stimulation; LH difference ratio = (LH level on MC3 – LH on trigger day)/LH level on MC3. The denominator for implantation rate is the number of embryos transferred. The denominator for ectopic pregnancy and miscarriage rate is the subjects of clinical pregnancies.*

## Discussion

This study is the first to identify that the high LH difference ratio induced by PPOS is associated with a higher rate of embryo implantation. Our data provide evidence suggesting that appropriate LH suppression before ovulation contributes to the optimal clinical outcome for patients with PCOS, such as improved implantation rate, clinical pregnancy rate, and live birth rate.

The PPOS regimen results in lower circulating LH levels in the follicular phase and requires a higher dosage of gonadotrophin than the conventional short protocol to obtain similar oocyte yields (Kuang et al., 2015; Wang et al., 2016; Zhu et al., 2016; Ata et al., 2020). This phenomenon indicates that the application of MPA during PPOS leads to stronger pituitary inhibition and deeper serum LH suppression (Kuang et al., 2015; Wang et al., 2016; Zhu et al., 2016; Massin, 2017; Huang et al., 2019; Ata et al., 2020). The high progestin environment also makes follicles less sensitive to gonadotropin stimulation (Zhu et al., 2016; Ata et al., 2020). Therefore, a higher amount of hMG was used to stimulate the growth of follicles in the PPOS group, which also resulted in a higher FSH level on the trigger day and FSH difference ratio in this study. However, other variables, such as hMG dose, FSH, or LH on trigger day, and FSH difference ratio showed no significant association with the implantation rate in logistic regression analysis.

We speculate that the higher live birth rate in the PPOS group in the current study is the result of better-quality embryos, since the influence of the hypoestrogenic milieu on the endometrium and other factors were balanced through the conditions of the freeze-all strategy, first FET cycle comparison and PSM (Huang et al., 2019). In general, embryonic implantation is a more reliable indicator of embryo quality and competency (Cha et al., 2012), excluding the disturbance from mother-infant and complicated obstetrical problems during pregnancy. Therefore, we selected implantation rate, rather than the live birth rate, as the dependent variable in logistic regression to identify potential influential factors. Our data revealed that a higher LH difference ratio might lead to a higher live birth rate in women with PCOS who underwent PPOS, indicating that lower circulating LH levels in the follicular phase might improve the oocyte competency and consequentially contribute to the success of embryo implantation. This finding was confirmed by both ROC analysis and the comparison of groups stratified according to the LH difference ratio. The high ratio group (LH difference ratio ≥0.385) had a higher percentage of patients treated with the PPOS protocol and better clinical outcome, while the low ratio group had a lower percentage of PPOS-treated patients and worse clinical outcome. Therefore, a strategy to predict the oocyte quality of patients with PCOS during follicle growth would be helpful for patients to receive a safe and effective ovarian stimulation. Here, we identified the characteristics associated with optimal responders with an LH difference ratio ≥0.385 by ROC.

Currently, there is no universal and established definition for oocyte competence. In general, oocyte competence is defined as the ability of a female gamete to mature into an egg that exhibits fertilization potential and develops to the blastocyst stage (Conti and Franciosi, 2018). Oocyte competence has also been defined based on the potential to sustain pregnancy and achieve a live birth (Palomba et al., 2017). In women with PCOS, oocyte competence is affected by extra- and intra-ovarian factors that influence the cumulus–oocyte interaction, oocyte maturation and embryonic development (Qiao and Feng, 2011). An elevated LH level during the follicle phase is considered to hamper oocyte development potential in humans as well as other species (Shoham et al., 1993). In our study, we investigated the endocrinological characteristics in ovary stimulation cycles corresponding to the transferred embryos. Even though high-quality embryos were transferred in FET cycles, the alterations in molecular factors originated from the different endocrine environments during the follicle growth and oocyte maturation might lead to different clinical outcomes.

Previous studies showed that tonic hypersecretion of LH during the follicular phase was associated with a significant decrease in the quality of both oocytes and embryos, resulting in reduced pregnancy rates and higher miscarriage rates among women with PCOS (Howles et al., 1986; Homburg et al., 1988; Tarlatzis and Grimbizis, 1997; van der Spuy and Dyer, 2004; Dumesic et al., 2008; Franks et al., 2008; Qiao and Feng, 2011). High LH levels may lead to abnormal granulosa cell function and induce oocyte atresia or prematuration (Howles et al., 1986; van der Spuy and Dyer, 2004; Dumesic et al., 2008; Franks et al., 2008). Several reports found that the addition of exogenous LH to the ovarian stimulation protocol may have negative effects on oocyte yield and quality when the level of endogenous LH ≥ 1 IU/l (Tesarik and Mendoza, 2002), and a higher LH exposure to the genital tract was found in non-pregnant patients, not in pregnant patients (Kolibianakis et al., 2003). And, other studies reported that the level of LH in patients with PCOS had no influence on oocyte and embryo quality, as these studies did not find any difference in clinical pregnancy rate even the LH levels varied among those women (Bosch et al., 2005; Doody et al., 2010; Weiss et al., 2019). In contrast, a study by Benmachiche et al. (2019) reported low serum LH levels on the day of GnRH-agonist trigger were associated with reduced rates of live birth. However, a recently published study of another center reported that low serum LH levels during ovarian stimulation with GnRH antagonist protocol had no impact on the live birth rate in freeze-all cycles (Luo et al., 2021). These inconsistent studies probably resulted from the variety in the definition of low LH, measurement parameters of LH, and the clinical interventions. These conflicting findings also indicated that the LH level may have an individual optimal value window during follicle growth, since the amount of LH necessary for standard follicle and oocyte maturation is still not known (Howles, 2000). In general, patients with PCOS show much higher basal LH levels than patients with non-PCOS infertility (Zhang et al., 2013). Our previous study showed that the clinical outcome was not related to the basal LH level in PPOS cycles (Sun et al., 2018). Thus, we chose the LH difference ratio as the main parameter in the current study, and the influence of basal LH level was excluded by adopting the PSM. Besides, we believed the LH difference ratio can reflect both the LH exposure and dynamic changes during the follicle growth, with an advantage with a single LH level on a certain day, either the start day or trigger day. Our data showed that PPOS suppressed LH level to a greater extent (with a higher LH difference ratio) than the short protocol in patients with PCOS, which might help maintain the appropriate LH level suitable for follicle growth and the development of high-quality oocytes. The difference in embryonic implantation potential in patients with PCOS may be from the differences in LH level suppression by various ovarian stimulation protocols. Moreover, the risk of congenital malformations is similar in both PPOS and other stimulation protocols, suggesting the safety of high progestin levels on developing follicles (Ata et al., 2020).

Although PPOS has been applied in patients with PCOS for IVF treatment in some clinical practices, to the best of our knowledge, this is the first study to demonstrate the association between the higher LH difference ratio induced by PPOS and the higher rate of implantation. We first provided the explanation of why PPOS shows a positive clinical outcome for patients with PCOS. The major limitation of our study are its retrospective nature, single control group, and therefore a randomized controlled trial or with other comparing stimulation protocols are needed to confirm our findings. More research is needed to clarify the underlying mechanism associated with the higher rate of implantation induced by the LH difference ratio. Moreover, a study with a long-term follow-up is important to investigate the safety of the PPOS protocol after birth.

## Conclusion

This study demonstrated that the PPOS protocol shows superior effects on embryo implantation, clinical pregnancy and live birth rate in the first FET cycles for patients with PCOS compared with the short protocol. We also present evidence showing that the increased LH difference ratio was associated with the improved clinical outcomes observed with PPOS, suggesting that maintaining the appropriate LH level during ovarian stimulation may contribute to optimal outcomes in patients with PCOS.

## Data Availability Statement

The raw data supporting the conclusions of this article will be made available by the authors, without undue reservation.

## Ethics Statement

The studies involving human participants were reviewed and approved by the Human Ethics Committee approval for human retrospective analysis came from the Institutional Review Board (IRB) of the Shanghai Ninth People’s Hospital. The patients/participants provided their written informed consent to participate in this study.

## Author Contributions

YK, HL, and QL conceived and designed this study. CC and SY contributed to the acquisition of data, analysis and interpretation of data, and manuscript drafting. WY, ZY, WJ, JS, ML, RC, DL, and LW participated in critical discussion of the results. QC contributed to the revision of the manuscript. All authors participated in the collection of data.

## Conflict of Interest

The authors declare that the research was conducted in the absence of any commercial or financial relationships that could be construed as a potential conflict of interest.

## Publisher’s Note

All claims expressed in this article are solely those of the authors and do not necessarily represent those of their affiliated organizations, or those of the publisher, the editors and the reviewers. Any product that may be evaluated in this article, or claim that may be made by its manufacturer, is not guaranteed or endorsed by the publisher.

## Supplementary Material

Supplementary Figure 1 The standardized differences before and after propensity score matching.

Supplementary Figure 2 Comparison of oocyte performance during the early developmental stage in ovarian stimulation cycles corresponding to the transferred embryos.
