# Supplementary figures and images for: Luteinizing Hormone Suppression by Progestin-Primed Ovarian Stimulation Is Associated With Higher Implantation Rate for Patients With Polycystic Ovary Syndrome Who Underwent in vitro Fertilization/Intracytoplasmic Sperm Injection Cycles: Comparing With Short Protocol
Source: Front Physiol. 2022 Feb 11;12:744968. doi: 10.3389/fphys.2021.744968 (PMC8874211; doi:10.3389/fphys.2021.744968)

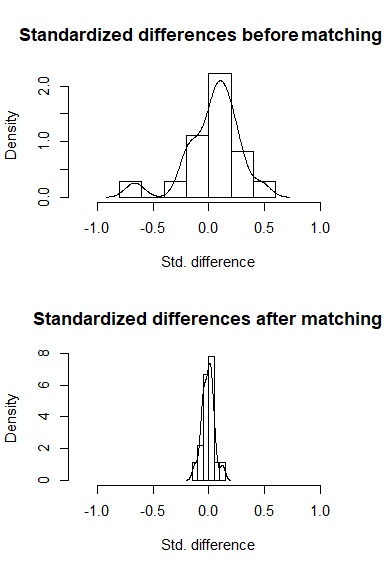

Supplement: Supplementary Figure 1 — The standardized differences before and after propensity score matching. [file Image_1.JPEG]

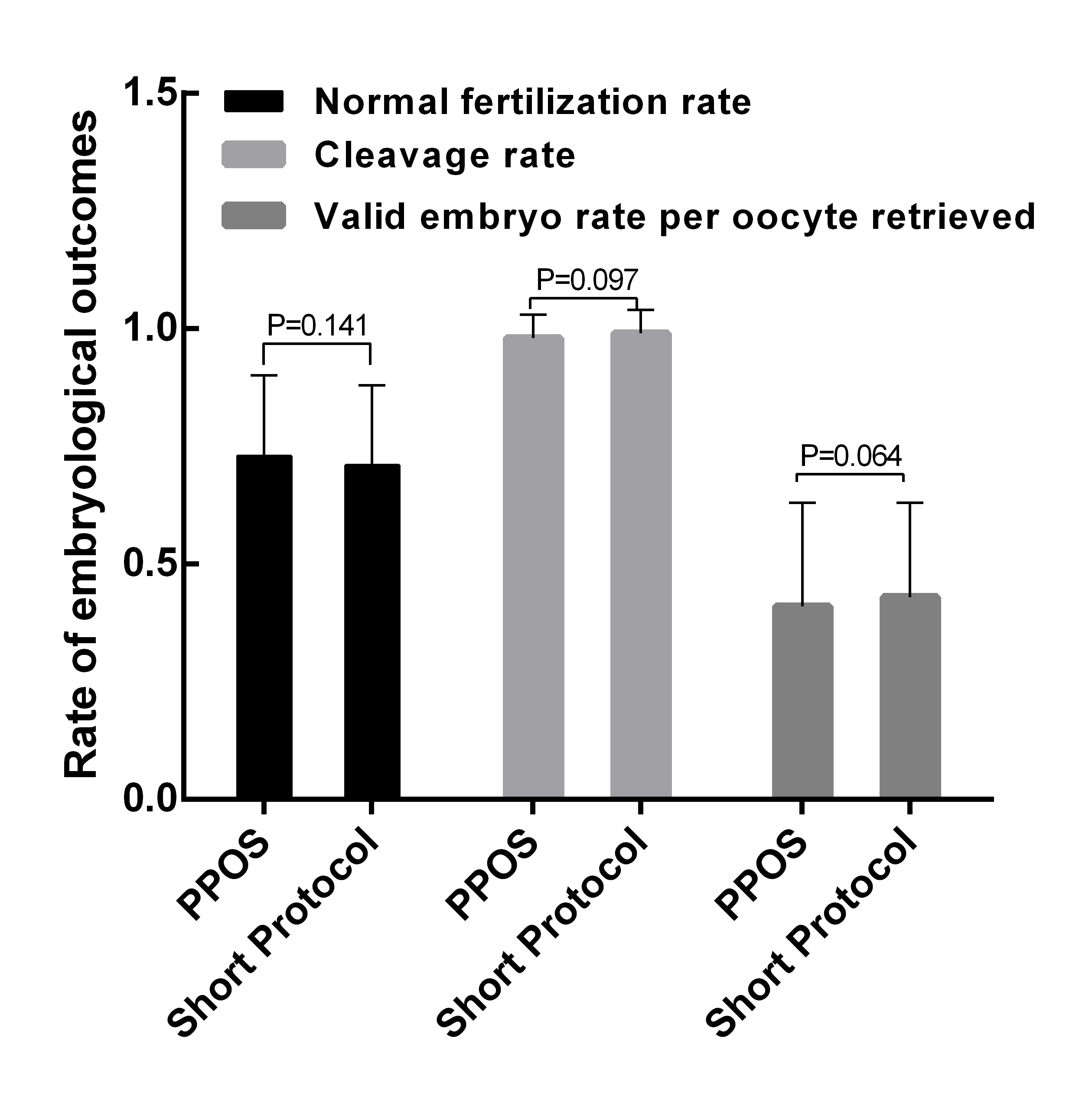

Supplement: Supplementary Figure 2 — Comparison of oocyte performance during the early developmental stage in ovarian stimulation cycles corresponding to the transferred embryos. [file Image_2.JPEG]
