# Supplementary material for: Luteinizing Hormone Suppression by Progestin-Primed Ovarian Stimulation Is Associated With Higher Implantation Rate for Patients With Polycystic Ovary Syndrome Who Underwent in vitro Fertilization/Intracytoplasmic Sperm Injection Cycles: Comparing With Short Protocol
Source: Front Physiol. 2022 Feb 11;12:744968. doi: 10.3389/fphys.2021.744968 (PMC8874211; doi:10.3389/fphys.2021.744968)
Supplement: Supplementary file 3 [file Data_Sheet_1.docx]

**Supplemental Table 1. Comparison of baseline characteristic of PCOS patients treated by PPOS or Short protocol before matching.**

|  | **PPOS**  **(*n*=1544)** | **Short protocol**  **(*n*=212)** | ***P* value** |
| --- | --- | --- | --- |
| **Age (years)** | 30.79 ± 4.91 | 31.38 ± 4.77 | 0.063 |
| **Body mass index (kg/m2)** | 23.21 ± 3.73 | 23.89 ± 4.35 | 0.118 |
| **Duration of infertility (years)** | 3.24 ± 3.83 | 4.03 ± 3.78 | 0.042 |
| **Gravidity** | 0(0,1) | 0(0,1) | 0.313 |
| **Parity** | 0(0,0) | 0(0,0) | 0.556 |
| **Primary infertility, n (%)** | 65.3(1008/1544) | 71.2(151/212) | 0.087 |
| **Infertility diagnosis, n (%)** |  |  | 0.055 |
| PCOS only/unknown factors | 23.8(367/1544) | 28.8(61/212) |  |
| PCOS + tubal factor | 52.4(809/1544) | 45.8(97/212) |  |
| PCOS + male factor | 13.5(208/1544) | 17.9(38/212) |  |
| PCOS + mixed factors | 10.4(160/1544) | 7.5(16/212) |  |
| **Basal endocrine profiles** |  |  |  |
| FSH (IU/L) | 5.68 ± 2.36 | 4.97 ± 2.53 | 0.107 |
| LH (IU/L) | 4.83 ± 4.11 | 5.08 ± 3.85 | 0.366 |
| E2 (pg/mL) | 33.31 ± 16.97 | 33.95 ± 29.57 | 0.002 |
| P (ng/mL) | 0.28 ± 0.22 | 0.25 ± 0.20 | 0.143 |
| T (ng/mL) | 0.40 ± 0.26 | 0.31 ± 0.16 | 0.101 |
| **Antral follicle count** | 19.23 ± 6.37 | 19.63 ± 8.52 | 0.511 |

Note: FSH, follicle-stimulating hormone; LH, luteinizing hormone; E2, estradiol; P, progesterone; T, testosterone.

**Supplemental Table 2.** **Main characteristics of maternal baseline and** **first frozen-thawed embryo transfer** **cycle by regimen group after matching.**

|  | **PPOS (*n*=304)** | **Short protocol (*n*=152)** | ***P* value** |
| --- | --- | --- | --- |
| **Age (years)** | 31.09 ± 3.41 | 31.18 ± 3.41 | 0.564 |
| **Body mass index (kg/m2)** | 23.49 ± 3.53 | 23.69 ± 4.15 | 0.872 |
| **Duration of infertility (years)** | 3.63 ± 2.57 | 3.95 ± 2.18 | 0.138 |
| **Gravidity** | 0(0,1) | 0(0,1) | 0.706 |
| **Parity** | 0(0,0) | 0(0,0) | 0.748 |
| **Primary infertility, n (%)** | 66.4(202/304) | 67.1(102/152) | 0.888 |
| **Infertility diagnosis, n (%)** |  |  | 0.947 |
| PCOS only/unknown factors | 28.9(88/304) | 29.6(45/152) |  |
| PCOS + tubal factor | 51.3(156/304) | 48.7(74/152) |  |
| PCOS + male factor | 12.8(39/304) | 13.8(21/152) |  |
| PCOS + mixed factors | 6.9(21/304) | 7.9(12/152) |  |
| **Basal endocrine profiles** |  |  |  |
| FSH (IU/L) | 5.15 ± 1.26 | 4.98 ± 1.51 | 0.404 |
| LH (IU/L) | 4.89 ± 2.97 | 4.97 ± 3.23 | 0.846 |
| E2 (pg/mL) | 33.40 ± 14.97 | 33.75 ± 29.46 | 0.073 |
| P (ng/mL) | 0.27 ± 0.14 | 0.26 ± 0.18 | 0.321 |
| T (ng/mL) | 0.39 ± 0.13 | 0.31 ± 0.06 | 0.310 |
| **Antral follicle count** | 19.46 ± 5.86 | 19.51 ± 7.39 | 0.978 |
| **Insemination method, n (%)** |  |  | 0.912 |
| IVF | 57.2(174/304) | 55.3(84/152) |  |
| ICSI | 24.7(75/304) | 26.3(40/152) |  |
| IVF + ICSI | 18.1(55/304) | 18.4(28/152) |  |
| **Endometrial thickness on FET day (mm)** | 11.67 ± 2.19 | 11.56 ± 2.47 | 0.638 |
| **Endometrial preparation, n (%)** |  |  | 0.643 |
| Mild stimulation | 51.6(157/304) | 49.3(75/152) |  |
| Hormone replacement therapy | 48.4(147/304) | 50.7(77/152) |  |
| **No. of embryos transferred, n (%)** |  |  | 0.713 |
| 1 | 15.8(48/304) | 14.5(22/152) |  |
| 2 | 84.2(256/304) | 85.5(130/152) |  |
| **Stage of embryos transferred, n (%)** |  |  | 0.629 |
| cleavage-stage | 85.9(261/304) | 87.5(133/152) |  |
| blast-stage | 14.1(43/304) | 12.5(19/152) |  |

Note: FSH, follicle-stimulating hormone; LH, luteinizing hormone; E2, estradiol; P, progesterone; T, testosterone; IVF, in vitro fertilization; ICSI, intracytoplasmic sperm injection; FET, frozen thawed embryo transfer.

**Supplemental Table 3.** **Comparison of clinical outcomes per transfer between PPOS and Short protocol in PCOS patients before matching.**

|  | **Embryo transfer cycles from PPOS (*n*=2489)** | **Embryo transfer cycles from Short protocol (*n*=361)** | | ***P* value** |
| --- | --- | --- | --- | --- |
| **Involved patient number** | 1544 | 212 | / | |
| **Type of embryo transfer cycles** |  |  | <0.001 | |
| ET | 0(0/2489) | 14.7(53/361) |  | |
| FET | 100(2489/2489) | 85.3(308/361) |  | |
| **No. of embryo transferred, n (%)** |  |  | 0.428 | |
| 1 | 17.5(435/2489) | 15.8(57/361) |  | |
| 2 | 82.5(2054/2489) | 84.2(304/361) |  | |
| **Stage of embryo transferred, n (%)** |  |  | 0.972 | |
| cleavage-stage | 84.7(2108/2489) | 84.8(306/361) |  | |
| blast-stage | 15.3(381/2489) | 15.2(55/361) |  | |
| **Implantation rate, n (%)** | 42.2(1916/4543) | 31.4(209/665) | <0.001 | |
| **Biochemical pregnancy rate per transfer, n (%)** | 63.6(1582/2489) | 51.8(187/361) | <0.001 | |
| **Clinical pregnancy rate per transfer, n (%)** | 58.6(1459/2489) | 45.4(164/361) | <0.001 | |
| **Ectopic pregnancy rate per transfer, n (%)** | 1.1(28/2489) | 0.3(1/361) | 0.134 | |
| **Miscarriage rate per transfer, n (%)** | 10.2(253/2489) | 9.4(34/361) | 0.660 | |
| **Live birth rate per transfer, n (%)** | 47.3(1178/2489) | 35.7(129/361) | <0.001 | |
| Singleton | 33.8(842/2489) | 27.1(98/361) |  | |
| Multiple | 13.5(336/2489) | 8.6(31/361) |  | |

Note: ET, embryo transfer; FET, frozen thawed embryo transfer.

**Supplemental Table 4. Comparison of endometrial preparation and embryo-transferred features between two groups re-divided by the level of LH difference ratio.**

|  | **Low group (N=158)** | **High group (N=298)** | ***P* Value** |
| --- | --- | --- | --- |
| **Endometrial thickness on FET day (mm)** | 11.52 ± 2.47 | 11.69 ± 2.18 | 0.398 |
| **Endometrial preparation, n (%)** |  |  | 0.565 |
| Mild stimulation | 46.8(74/158) | 49.7(148/298) |  |
| Hormone replacement therapy | 53.2(84/158) | 50.3(150/298) |  |
| **No. of embryos transferred, n (%)** |  |  | 0.088 |
| 1 | 11.4(18/158) | 17.4(52/298) |  |
| 2 | 88.6(140/158) | 82.6(246/298) |  |
| **Stage of embryos transferred, n (%)** |  |  | 0.882 |
| cleavage-stage | 86.1(136/158) | 86.6(258/298) |  |
| blast-stage | 13.9(22/158) | 13.4(40/298) |  |

Note: FET, frozen thawed embryo transfer; LH difference ratio= (LH on MC3-LH on trigger day)/ LH on MC3.
